# A mosaic form of microphthalmia with linear skin defects

**DOI:** 10.1186/s12887-018-1234-4

**Published:** 2018-08-01

**Authors:** Nina Prepeluh, Bojan Korpar, Andreja Zagorac, Boris Zagradišnik, Andreja Golub, Nadja Kokalj Vokač

**Affiliations:** 1 Laboratory of Medical Genetics, University Medical Centre Maribor, Ljubljanska 5, 2000 Maribor, Slovenia; 2 Department of Perinatology, Division of Gynaecology and Perinatology, University Medical Centre Maribor, Maribor, Slovenia; 3 Medical Faculty, University of Maribor, Maribor, Slovenia

**Keywords:** Microphthalmia, Linear skin defects, Mosaicism, Xp22.31p22.2 deletion, *HSSC* gene

## Abstract

**Background:**

Microphthalmia with linear skin defects (MLS) syndrome is a rare neurodevelopmental X-dominant disorder. It presents in females as it is normally lethal in males. Three causative genes for MLS syndrome (OMIM 309801) have been identified all taking part in mitochondrial respiratory chain and oxidative phosphorylation. In our case, we describe a newborn with mosaic deletion encompassing *HCCS* gene resulting in unilateral microphthalmia and facial skin lesions.

**Case presentation:**

A girl was born with caesarean section at 40 weeks of gestation. Clinical findings revealed anophthalmia of the left eye. The left eyelids were intact, the orbit was empty and the right eye was normal, without any abnormalities. She had typical linear skin defects on the left cheek, one on the left side of the neck, and two on the 3th and 4th fingers of the left hand. The other clinical findings and the neurological exam were normal. US of the brain and EEG were normal. Molecular karyotyping using BlueGnome CytoChip Oligo 4× 180K array was performed detecting an approximately 18% mosaic 3.3 Mb deletion (arr[GRCh37] Xp22.31p22.2(8,622,553_11,887,361)× 1[0.18]). FISH using RPCI11-768H20 BAC clone on cultivated interphase and metaphase lymphocytes was used to confirm the array results. The observed deletion was present in 29% of cells (46,XX,ish del(p22.2p22.31)(RPCI11-768H20)[60/205]).

**Conclusions:**

In this report we present a female proband with MLS syndrome. To our knowledge, there have been only few other cases of mosaic MLS syndrome described in the literature. Our case shows that low grade mosaicism does not preclude full clinical presentation and further supports the critical role of the X inactivation pattern in the development of the clinical findings.

## Background

Microphthalmia with linear skin defects (MLS) syndrome is a rare X-linked dominant disorder affecting mainly the eyes and the skin. It affects only girls as it is lethal in males. The characteristic features of the syndrome are microphthalmia (sometimes anophthalmia) that may be uni- or bilateral and linear skin defects, which are usually limited to the face and neck. The accompanying abnormalities include other ocular abnormalities, structural neurological abnormalities, developmental delay, congenital heart defects and short stature. The disorder shows high phenotypic variability among affected individuals including between family members [1–3].

To date, less than a hundred patients with MLS syndrome have been reported. The syndrome is most often a result of a de novo pathogenic mutation, although rare familial cases have been described [3–5]. Genetic abnormalities resulting in Xp22.2 monosomy as well as some intragenic deletions and point mutations in *HCCS* and *COX7B* genes were found to be responsible for the syndrome. Recently a mutation in *NDUFB11* gene was reported as causative in two cases. All three genes encode proteins that are essential for proper function of mitochondrial respiratory chain (MRC) and oxidative phosphorylation [6–8]. Therefore, it is possible that the impaired oxidative phosphorylation and its consequences (e.g. lack of ATP, abnormal production of free oxygen radicals) are responsible for the clinical manifestations observed in MLS syndrome [8].

In this report we present the clinical and molecular data of an infant girl with mosaic deletion of the chromosome Xp22.2. The deletion encompasses 12 OMIM genes, including *HCCS* gene, and is present in approximately 18% of the cells. Despite the mosaic form of MLS syndrome, the girl presents with the fully expressed clinical presentation.

## Case presentation

The female proband is the first child of healthy parents. None of the parents showed any dysmorphic signs and their family history was unremarkable. An infant girl was born with elective caesarean section at 40 weeks of gestation. Birth weight was 3640 g, birth length 50 cm, head circumference 35 cm and the Apgar scores were 9/9/9. Mother had gestational diabetes mellitus controlled by diet and was treated with beta blockers in the last month of pregnancy because of paroxysmal tachycardia.

**Fig. 1:**
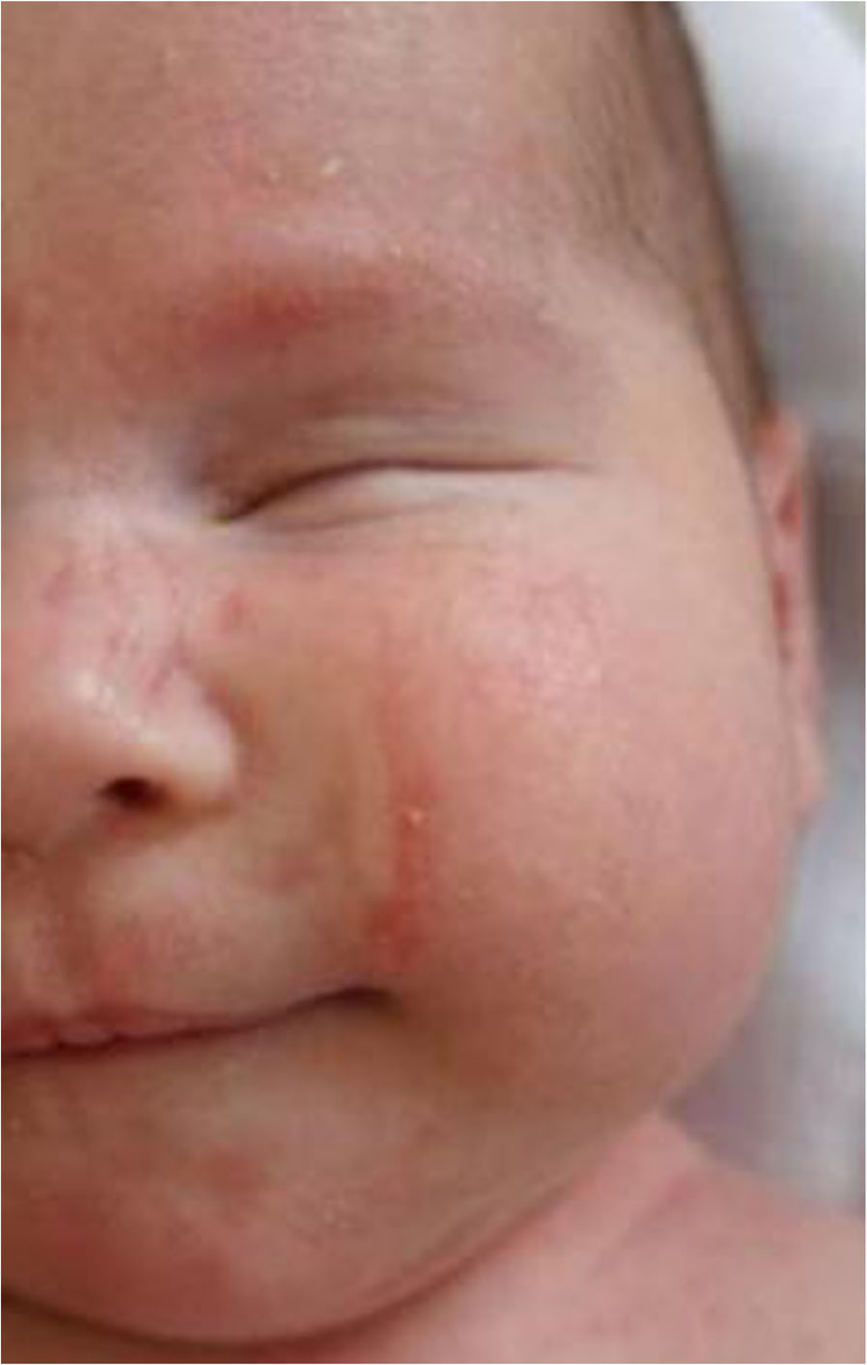
An infant girl with left-sided anophthalmia and skin defect on the cheek

At birth the newborn female showed linear skin defects on the left cheek, one on the left side of the neck and two on the 3th and 4th fingers of the left hand. Ophthalmologic evaluation revealed left-sided anophthalmia with intact left eyelids (Fig.1). The right eye was without any abnormalities. Echocardiography detected atrial septal defect and patent ductus arteriosus, which was not hemodynamically important. Other clinical findings and neurological exam were normal. Brain US and EEG were also normal.

The clinical diagnosis of MLS syndrome was suspected and a blood sample was taken for microarray analysis.

At the follow-up two months later, the skin defects were almost completely healed and only barely seen.

### Genetic analysis

**Fig. 2:**
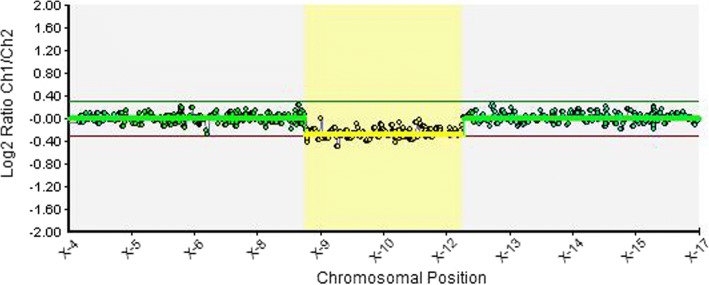
Array CGH of the patient revealed deletion of Xp22.2 in mosaic form: arr[GRCh37]Xp22.31p22.2(8622553–11,887,361)× 1[0.18]

Molecular karyotyping using the BlueGnome CytoChip Oligo 4× 180K array was performed following the protocol. The array CGH analysis demonstrated an approximately 18% mosaic 3.3 Mb deletion of Xp22.31p22.2 (arr [GRCh37] Xp22.31p22.2(8622553–11,887,361)× 1[0.18]) (Fig.2). The deletion encompasses 12 OMIM genes, including *KAL1, FAM9A, FAM9B, TBL1X, GPR143, SHROOM2, CLCN4, MID1, HCCS, ARHGAP6, AMELX* and *MSL3*.

**Fig. 3:**
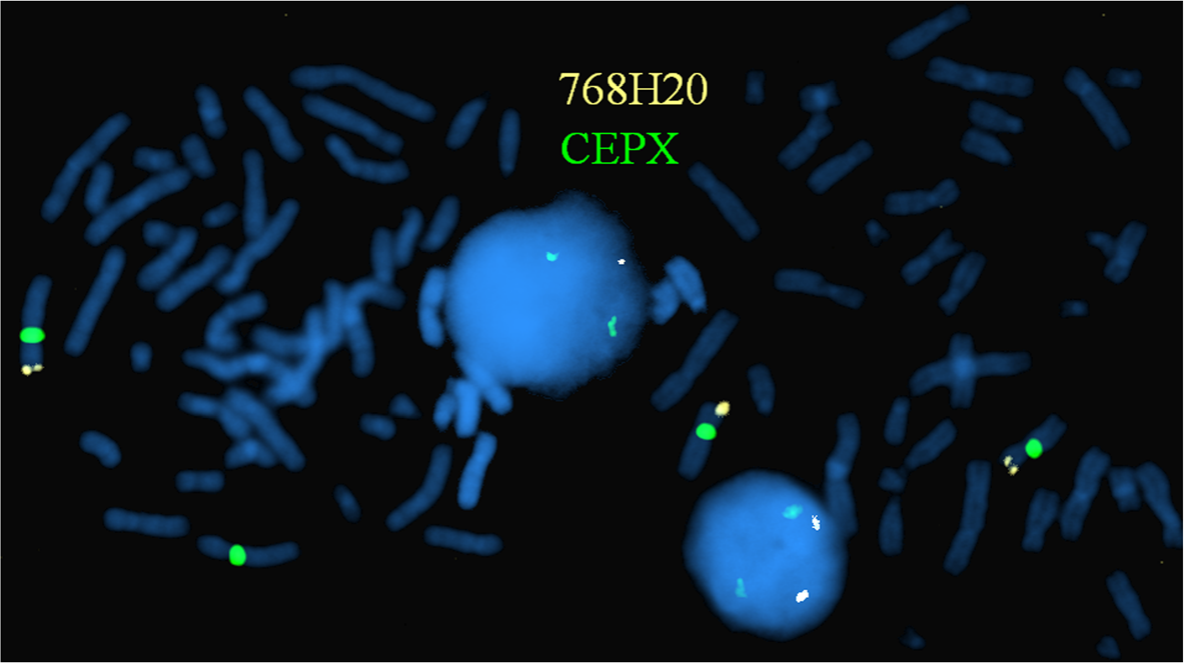
FISH analysis with RPCI11-768H20 (gold) and CEPX (green) DNA probes on double metaphase spreads and interphase nuclei. One metaphase and one nuclei with deletion of RPCI11-768H20 probe on X chromosome and another with two normal X chromosomes and two signals for RPCI11-768H20 probe, demonstrating mosaicism

To confirm the array results, FISH using BAC probe RPCI11-768H20 on cultivated lymphocytes was used. Mosaicism was confirmed in 29% of cells by producing only one hybridisation signal (of the normal chromosome X) instead of two (Fig.3).

X chromosome inactivation (XCI) pattern was performed by examining the methylation status of the AR1 and AR4 loci using a standard protocol. A ratio 92:8 XCI was detected.

## Discussion and conclusion

In this paper we report an infant girl with a mosaic interstitial Xp22.2 deletion. A newborn presented with typical clinical signs of MLS syndrome – congenital linear skin defects and left-sided anophthalmia, accompanied by congenital heart defects. The latter is known to be an associated feature of the syndrome and represents one of the so called “minor criteria of MLS syndrome”.

The syndrome was firstly reported to be caused by genetic abnormalities resulting in Xp22.2 monosomy. With technological advances we detected pathogenic mutations in three critical genes encoding proteins important in mitochondrial respiratory chain and oxidative phosphorylation – *HCCS, COX7B* and *NDUFB11* genes. Although the exact pathophysiological mechanism of disease is not known yet, it is hypothesised that cells with the mutated X chromosome active express impaired oxidative phosphorylation which leads to cell dying and development of MLS- associated features [8, 9].

The disorder may present with a broad spectrum of congenital anomalies. The majority of patients show classical eye and skin abnormalities, but the clinical presentation may vary widely across patients and even within the family members [1–4, 10, 11]. Van Rahden et al. reported of six patients who all presented with microphthalmia, two had accompanied anophthalmia and five had sclerocornea. Linear skin defects were found in four patients while were other abnormalities limited to single cases [3]. Wimplinger et al. described a familial form of MLS syndrome showing high clinical variability between patients. They reported of a mother and two daughters who had intragenic deletion in *HCCS* gene. While mother was asymptomatic and one daughter showed only ocular abnormalities, the youngest one presented with left-sided anophthalmia, sclerocornea and skin defect [2]. Interestingly, although no clear genotype-phenotype correlations have been made so far, it seems that there are some variations in clinical picture according to the mutated gene [3, 10, 12]. Indrieri et al. described four patients with MLS syndrome caused by *COX7B* mutations, who show only linear skin defects and lack of ocular involvement. This is in contrast to *HCCS* mutations which normally affect both the eye and skin as is seen in our case [7].

The mechanism responsible for high phenotypic variability observed is currently unknown, although X inactivation patterns and presence of mosaicism are thought to play important role [2–4, 9, 13]. Cells with active abnormal X chromosome are thought to die earlier which leads to the predominance of the cells with active wild-type X chromosome, skewed XCI and milder phenotype [3, 13]. To date only few cases of mosaic form of MLS syndrome have been described. Wimplinger et al. reported a girl with classical signs of MLS syndrome and her asymptomatic mother who presented only with short stature and low posterior hairline. It was found that both carry terminal Xp deletion (mother in mosaic form - 45,X [11]/46,del(X)(p22.2)[89]) and have a completely skewed X inactivation. It was concluded that both mechanisms - the somatic mosaicism and non-random X- inactivation pattern in favour of non-mutated X chromosome might contribute to the lack of symptoms in the mother [9]. On the contrary, Ogata et al. reported a girl with a complex karyotype and a deletion of Xp22 presented in 58% of cells. She presented with right-sided microphthalmia, sclerocornea, skin defects and absent corpus callosum. The full blown clinical presentation was hypothesised to be the consequence of inactivation of the normal X chromosome [14]. Similarly, our proband girl presented with fully expressed clinical symptoms despite low grade mosaicism and apparently skewed XCI detected in her blood cells. One possible explanation for the clinical presentation observed is that we tested only blood cells so the percent of cells with a deletion of Xp22 among other tissues is not known. It is possible that the percent of the mutated cells among blood cells is under-represented due to a negative selection. Furthermore, taking into consideration that the mutation was presented only in 18–29% of cells, we interpret the detected XCI ratio 92:8 in blood cells as partially by chance, which also contribute to the explanation the clinical presentation. The XCI pattern in other tissues is however not known. Taking together, we think that partially random X inactivation pattern and the possibility of the higher percent of cells with the deletion of Xp22 among critical tissues play the pivotal role in developing the full-blown clinical picture observed in our case.

To sum up, in this report we present an infant girl with mosaic deletion of Xp22.2 and a fully expressed MLS syndrome despite low grade mosaicism detected. To our knowledge, this case presents with the lowest mosaicism reported in association with MLS syndrome. It shows that the mosaicism does not preclude the full clinical expression and supports the critical role of X inactivation pattern in the development of clinical presentation.

## Abbreviations

ATP: Adenosine triphosphate
BAC: Bacterial artificial chromosome
CGH: Comparative genomic hybridization
EEG: Electroencephalogram
FISH: Fluorescent in situ hybridization
GRCh37: Genome Reference Consortium human genome (build 37)
MLS: Microphthalmia with linear skin defects syndrome
OMIM: Online Mendelian Inheritance in Man
RPCI: Bacterial Artificial Chromosome Library
US: Ultrasound
XCI: X chromosome inactivation
